# 

**Published:** 1862-09

**Authors:** 


### CONTENTS.

### ORIGINAL CONTRIBUTIONS.

ART. XXVI. A Case of Masturbation, with Remarks. By E. H. Neyman, M.D., Cedar Bluffs, Iowa, .......... 523
ART. XXVII. Army Reports. From H. Wardner, M.D., Brigade-Surgeon, .......... 531
ART. XXVIII. Cases of Gunshot Wound. Reported by O. B. Ormsby, Assistant-Surgeon 18th Reg't. Ill. Vols., .......... 535
ART. XXIX. Clinical Remarks on the Bowel-Affections of Children in the Medical Wards of the Mercy Hospital. By N. S. Davis, M.D., Professor of Clinical Medicine, etc., ...... 538

### SELECTIONS.

Report of the Committee of the Royal Medical and Chirurgical Society, on the Subject of Suspended Animation, .......... 545
On the Treatment of Tubercular Hemoptysis. By Dr. Wm. Brinton, Physician to St. Thomas's Hospital, .......... 553
Clinical Remarks on Sympathetic Inflammation of the Eyeball, its Danger and the Means of Arresting it. Made at the Central London Ophthalmic Hospital. By Haynes Walton, Surgeon to the Hospital, .......... 557
Some of the Effects of Tobacco. By P. J. Farnsworth, M.D., Lyons, Io. 560
The Obstetric Bag. A Description of the Instruments used in Operative Midwifery. A Lecture delivered in St. Thomas's Hospital, by Robert Barnes, M.D., etc., etc., .......... 563
Dangerous Fashionable Folly—Green Artificial Flowers, .......... 537

### EDITORIAL.

Medical Department of Lind University, .......... 571
"Is Alcohol Food?" .......... 572
Resignations of Army Surgeons, .......... 576
Artificial Limbs for Soldiers, .......... 576
Death of Dr. John C. Cheeseman, .......... 577
Hospital Inspection, .......... 577
Note from R. Roskoten, M.D., .......... 577
Physician's Hand-Book of Practice for 1863: by William Elmer, M.D. New York: W. A. Townsend, publisher, 39 Walker Street, .......... 577
Physician's Pocket Memorandum: for 1863. By C. H. Cleveland, M.D. Cincinnati; Bradley & Webb, Printers, .......... 578
Experiments on the Formation of Infusoria in Boiled Solutions of Organic Matter, enclosed in Hermetically Sealed Vessels, and Supplied with Pure Air, .......... 578
Inversion of the Uterus occurring Spontaneously Eighty Hours after Delivery, .......... 580
Researches on the Influence of Culinary Salt and Coffee on the Metamorphosis of Tissue, .......... 581
Varicose Ulcers of the Leg, .......... 582
The Liver in Cholera Infantum, .......... 583
Medical Council of Great Britain, .......... 584
A Dollar that Pays Well, .......... 584

